# Effect of Fermentation Conditions on Functional Quality of Napa Cabbage Kimchi

**DOI:** 10.3390/foods14162826

**Published:** 2025-08-15

**Authors:** Jaecheol Kim, Hyosun Park, BoKyung Moon, Suna Kim

**Affiliations:** 1 Food and Nutrition Major, School of Bio-Health Convergence, College of Natural Science, Sungshin Women’s University, Seoul 01133, Republic of Korea; jckim@sungshin.ac.kr; 2 Department of Food and Nutrition, College of Biotechnology and Natural Resources, Chung-Ang University, Anseong 17546, Republic of Korea; park318402@naver.com (H.P.); bkmoon@cau.ac.kr (B.M.); 3 Division of Human Ecology, College of Natural Science, Korea National Open University, Seoul 03087, Republic of Korea

**Keywords:** kimchi, fermentation, napa cabbage, lactic acid bacteria, glucosinolates, ascorbigen

## Abstract

This study investigated the effects of storage temperature on the functional quality of kimchi during short- and long-term fermentation. Pretreated napa cabbage (NC) quality was analyzed, and kimchi was prepared using pretreated NC and stored at either 4 °C or 15 °C until reaching optimal and excessive fermentation stages. Fermented kimchi samples were analyzed for pH, lactic acid bacteria (LAB) counts, total polyphenol content (TPC), DPPH and ABTS radical scavenging activities, and glucosinolates and their breakdown products. Fermentation at 15 °C progressed rapidly, reaching the optimal pH range (4.0–4.5) within 3 days, and resulted in significantly higher LAB counts and TPC compared to samples stored at 4 °C (*p* < 0.05). In contrast, prolonged storage at 4 °C led to a decrease in both TPC, radical scavenging activities, and LAB counts during the excessive fermentation stage. Glucosinolates were progressively degraded overtime; however, their breakdown product, ascorbigen, increased under short-term fermentation at 15 °C than during prolonged storage at 4 °C. These results suggest that short-term fermentation at 15 °C is more effective than long-term fermentation at 4 °C in enhancing the functional quality of kimchi by promoting LAB growth and preserving bioactive compounds.

## 1. Introduction

Kimchi is a major Korean fermented vegetable dish. During fermentation, the counts of beneficial lactic acid bacteria, including *Lactobacillus brevis, Lb. fermentum, Lb. plantarum, Leuconostoc mesenteroides, and Weissella confusa*, and the concentrations of bioactive compounds increase markedly, which is known as a health-promoting super food worldwide [1]. *Kimjang*, the communal preparation and storage of kimchi for winter, remains a cornerstone of Korean culinary tradition. Traditionally, kimchi was stored at low temperature throughout the cold season and consumed gradually over several months. However, the year-round availability of commercial kimchi has become commonplace [2,3]. Consequently, there is growing demand for novel ripening conditions and processing techniques that enable rapid production of high-quality kimchi for reliable year-round supply.

Kimchi fermentation is highly sensitive to storage temperature. At low temperatures (e.g., 4 °C), the fermentation rate slows, whereas at room temperatures (e.g., 15–20 °C), ripening proceeds rapidly [4]. However, excessive fermentation can cause a deterioration of texture and loss of functional components [5]. These issues highlight the need for more advanced storage and processing methods to optimize ripening and maintain the quality of kimchi. Although sensory properties, pH, and lactic acid bacteria (LAB) counts are commonly used to assess the fermentation status and quality of kimchi, additional indicators—such as total phenolic content (TPC), antioxidant activities (DPPH and ABTS), glucosinolates and their metabolites, and LAB composition—also serve as useful markers of its functional quality. Among them, glucosinolates, found in abundance in napa cabbage and radish, are converted during kimchi fermentation into metabolites such as ascorbigen and isothiocyanates, which exhibit anticancer, antioxidant, and anti-inflammatory properties [6,7]. Kimchi is commonly distributed under refrigerated conditions, and 4 °C has therefore been widely adopted as its storage temperature. Some studies at this temperature have examined changes in the LAB profile along with microbially derived metabolites, volatile compounds, and other metabolic components [8,9,10,11,12]. However, changes in glucosinolates and their metabolites during long-term storage or higher storage temperatures have not been reported as far as we know. In addition, few studies have investigated how pretreatment methods, such as cutting or salting prior to fermentation, affect the initial quality of napa cabbage as a major ingredient in kimchi.

This study aimed to evaluate how pretreatment of napa cabbage (whole, cut, and salted) and storage temperature (4 °C and 15 °C) affect the functional quality of kimchi—specifically total phenolic content, antioxidant activity, glucosinolate metabolism, and lactic acid bacterial change—during short- and long-term fermentation.

## 2. Materials and Methods

### 2.1. Chemicals

Methanol used for extraction was purchased from Junsei Chemical Co., Ltd. (Saitama, Japan), and acetonitrile for LC analysis was obtained from J.T. Baker (Avantor Co., Radnor, PA, USA). Standards of neoglucobrassicin (NGB), 4-methoxyglucobrassicin (4MGB), glucobrassicin, glucoraphenin, glucoraphanin, glucoraphasatin, glucoerucin, progoitrin, and gluconapin were purchased from Extrasynthese (Genay, France), while sulforaphane and ascorbigen were obtained from Biosynth Carbosynth (Berkshire, UK). All other chemicals were purchased from Sigma Chemical Co. (St. Louis, MO, USA).

### 2.2. Preparation of Kimchi

#### 2.2.1. Kimchi Ingredients

Whole napa cabbage (WNC, Korean cabbage), weighing approximately 3 to 3.5 kg per head, was used to prepare kimchi. The ingredients, including radish, green onion, garlic, ginger, red pepper powder (Yeongwol Nonghyup Corp., Yeongwol-gun, Gangwon-do, Republic of Korea), sun-dried salt, sugar, and anchovy fish sauce (CJ, Jung-gu, Seoul, Republic of Korea), were purchased from the local market.

#### 2.2.2. Pretreatment of Napa Cabbage for Kimchi

Pretreatment of napa cabbage for kimchi was conducted using a modified method from a previous study [13,14]. To prepare the kimchi, the outer contaminated leaves of napa cabbage were removed, and the remaining leaves were separated individually. Based on leaf length and position, they were classified into three groups: outer, middle, and inner leaves. After recording the weight of each group, a total of 1 kg of leaf samples was collected by combining equal amounts from each group. The outer and middle leaves were cut into 4 cm lengths and further divided into three subgroups: white portion, white portion with partial green leaf, and green leaf. The inner leaves were divided into two subgroups: white portion and green leaf. In total, eight subgroups were obtained, and the weight of each subgroup was measured. A 1 kg mixture sample, consisting of equal portions from each subgroup, was prepared for analysis (CNC, cut napa cabbage).

#### 2.2.3. Salting of NC

A 10% brine solution was prepared using sea salt. Brine, at twice the weight of napa cabbage from each subgroup, was added to polyethylene bags containing samples. After air removal, the bags were sealed, and the brining was conducted for 10 h. To ensure uniform salt penetration, the bags were shaken every 2 h. Following the brining, the napa cabbage parts were rinsed twice with tap water and once with distilled water and then dehydrated for 2 h. The weight after dehydration was recorded. The brined napa cabbage from each subgroup was equally divided by group, and 1 kg pooled samples were prepared. One 1 kg sample was used for compositional analysis (SNC, salted napa cabbage), and the remaining brined NC was used for kimchi preparation.

#### 2.2.4. Sample Preparation and Storage

WNC, CNC, and SNC samples were freeze-dried immediately after collection, except for 200 g of each, which was reserved for pH and LAB analysis. The freeze-dried powders were stored at –75 °C until further analysis.

The kimchi was prepared by mixing 1 kg of pooled brined napa cabbage (SNC) with 130 g of radish, 20 g of green onion, 14 g of garlic, 6 g of ginger, 35 g of red pepper powder, 10 g of sugar, and 22 g of anchovy fish sauce. The final salt content of the prepared kimchi was 2.3%. The mixture was packed into bags made of LDPE and LLDPE film. After air removal, the bags were sealed and placed in airtight containers (LocknLock Co., Seoul, Republic of Korea). After standing at room temperature for 1 h, kimchi samples were collected to represent the initial stage (non-fermented kimchi, NFK). The remaining samples were stored at 4 °C to obtain optimally and excessively fermented kimchi at 4 °C (OFK04 and EFK04, respectively) and at 15 °C in a low-temperature incubator to obtain optimally and excessively fermented kimchi at 15 °C (OFK15 and EFK15, respectively). Sampling of long-term storage kimchi was performed at approximately one-week intervals, scheduled according to researcher availability.

### 2.3. pH Measurement

To determine the optimal fermentation period, the pH of kimchi was measured periodically during storage. A 100 g sample was uniformly collected from different parts of the kimchi and homogenized using a hand blender (Model 4191, Braun, Hesse, Germany). The homogenate was filtered through four layers of gauze to extract the juice. The pH of the extracted juice was measured at room temperature using a pH meter (ORION STAR A211, Thermo Scientific, Waltham, MA, USA).

### 2.4. Enumeration of Leuconostoc *spp.* and Lactobacillus *spp.*

A 25 g sample of kimchi was placed into a sterile stomacher bag and diluted 10-fold with 0.1% sterile peptone solution. The sample was homogenized using a stomacher, and serial 10-fold dilutions were subsequently prepared with the same diluent. Aliquots of the diluted samples were plated onto *Lactobacilli* MRS agar supplemented with 0.002% bromophenol blue and incubated at 37 °C for 48 h. After incubation, colony-forming units (CFU) were counted and multiplied by the dilution factor to determine bacterial populations. Colonies appearing dark blue on BPB-MRS agar were counted as *Leuconostoc* spp., while colonies that were light blue or had a dark blue halo in the center were identified as *Lactobacillus* spp. [15,16].

### 2.5. UPLC-MS/MS Analysis

Here, 1 g of freeze-dried sample was mixed with 20 mL of methanol. The mixture was vortexed for 1 min and kept in a 60 °C water bath (BS-11, Lab Companion, Seoul, Republic of Korea) for 60 min. The mixture was vortexed again for 1 min and centrifuged at 1500× *g* for 10 min. The supernatant was filtered through a 0.2 μm syringe filter (DISMIC-13JP; Advantec, Tokyo, Japan). Glucosinolates and their breakdown products were analyzed using a modified method of a previous study [17]. GLSs and their breakdown products were analyzed using an ACQUITY UPLC H-class coupled with an Xevo TQD (triple quadrupole mass spectrometer) (Waters, Milford, MA, USA) that was equipped with an ACQUITY HSS T3 column (100 mm × 2.1 mm, 1.8 μm; Waters). Detailed UPLC-MS/MS and multiple reaction monitoring parameters are shown in Tables S1 and S2.

### 2.6. Total Polyphenol Content

A 1 g portion of freeze-dried sample was mixed with 10 mL of 80% methanol and extracted using a sonicator (Model 8510, Branson, Danbury, CT, USA) for 1 h. The extract was centrifuged at 2700× *g* for 5 min at 20 °C, and the supernatant was collected and filtered through filter paper (No. 2, Advantec Korea, Seoul, Republic of Korea). The filtrate was concentrated under nitrogen in a 60 °C water bath and freeze-dried. The dried extract was weighed to determine its solid content and re-dissolved to 80% methanol. After filtration through a 0.45 μm syringe filter, a stock solution (200 mg/mL) was prepared for analysis.

Total polyphenol content (TPC) was determined using a modified method of previous studies [18,19]. 250 μL of extract (10 mg/mL) was mixed with 250 μL of Folin–Ciocalteu’s phenol reagent and incubated at room temperature for 3 min. Then, 750 μL of 2% Na_2_CO_3_ solution was added, and the mixture was allowed to react in the dark for 2 h. Absorbance was measured at 760 nm using a spectrophotometer (OPTIZEN α, Mecasys, Daejeon, Republic of Korea) and a 10 mm quartz cuvette (Hellma, Müllheim, Germany). TPC was expressed as gallic acid equivalents (GAE) per gram of dry sample, based on a calibration curve constructed with gallic acid concentrations ranging from 6.25 to 200 μg/mL.

### 2.7. Radical Scavenging Activities

Kimchi extracts prepared as described in Section 2.6 were used to analysis radical scavenging activities. Radical scavenging activities were determined using a modified method of previous studies [18,19].

To determine DPPH radical scavenging activity, 100 μL of sample extract was mixed with 900 μL of 100 μM DPPH solution and incubated in the dark at room temperature for 30 min. The absorbance was then measured at 517 nm using a spectrophotometer (OPTIZEN α, Mecasys, Daejeon, Republic of Korea) with a 10 mm quartz cuvette (Hellma, Müllheim, Germany). DPPH radical scavenging activity (%) was calculated using the following equation:

DPPH radical scavenging activity (%) = [1 − Absorbance of control/Absorbance of sample] × 100

For ABTS radical scavenging activity, a stock solution was prepared by mixing equal volumes of 7 mM ABTS and 2.45 mM potassium persulfate in distilled water, followed by incubation in the dark at room temperature for 24 h to generate the ABTS·^+^ radical cation. The resulting solution was diluted with ethanol to obtain an absorbance of 0.70 ± 0.02 at 734 nm. For the assay, 990 μL of the diluted ABTS·^+^ solution was mixed with 10 μL of sample extract and reacted in the dark at room temperature for 5 min. The absorbance was measured at 734 nm using a spectrophotometer (OPTIZEN α, Mecasys, Daejeon, Republic of Korea) and a 10 mm quartz cuvette (Hellma, Müllheim, Germany). ABTS radical scavenging activity (%) was calculated using the following formula:

ABTS radical scavenging activity (%) = [1 − Absorbance of control/Absorbance of sample] × 100

### 2.8. Statistical Analysis

All analytical experiments were conducted in triplicate, and the mean and standard deviation were calculated based on the dry weight. Statistical analysis was conducted by one-way analysis of variance (ANOVA) with Duncan’s multiple range test (*p* < 0.05) using IBM SPSS 26.0 software (IBM Corp., Armonk, NY, USA).

## 3. Results and Discussion

### 3.1. Effect of Pretreatment Methods on Napa Cabbage Quality

#### 3.1.1. TPC and LAB Counts of Pretreated Napa Cabbages

TPC and Log CFU values of *Leuconostoc* spp. and *Lactobacillus* spp. in differently pretreated NC samples are shown in Table 1. Among the samples, CNC had the highest TPC (3.80 ± 0.05 mg GAE/g DW), followed by WNC and SNC. While there was no significant difference between CNC and WNC, the TPC in SNC was significantly lower than that of the other two samples (*p* < 0.05). Chung et al. (2021) reported that the polyphenol content in salted cabbage was lower than that in fresh cabbage [20]. Similarly, Chun et al. (2004) found that the TPC of sauerkraut made from salted cabbage was lower than that of raw cabbage [21]. Cvetković et al. (2019) also demonstrated that osmotic treatment using a salt and sugar mixture led to the loss of certain phenolic compounds, such as sinapic acid, and bioactive compounds, including ascorbic acid [22]. Consistent with these previous studies, the lower TPC observed in SNC may be attributed to the osmotic effect of salt, which facilitates the leaching of phenolic compounds and other water-soluble bioactives during the salting process.

*Leuconostoc* spp. were detected only in WNC (3.05 ± 0.13 log CFU), whereas *Lactobacillus* spp. were not detected in CNC. *Lactobacillus* spp. counts in WNC and SNC had no significant difference (*p* < 0.05). The absence of *Leuconostoc* spp. in CNC and SNC may be attributed to the loss of surface-associated microorganisms during pretreatment processes such as washing. In addition, the initial salting condition (10% salt brine) may have been too high compared to the optimal salinity for *Leuconostoc* growth, which is typically 2–3% [23,24]. In contrast, although *Lactobacillus* spp. were not detected in CNC, they were present in SNC. This may be explained by the greater halotolerance of *Lactobacillus* spp. compared to *Leuconostoc* spp., allowing for their partial survival under high-salinity conditions. Consistent with results in this study, previous studies also reported that certain LAB species may not be detected during the early salting stage of cabbage, although *Lactobacillus* spp. are often detectable [24,25].

#### 3.1.2. DPPH and ABTS Radical Scavenging Activities of Pretreated Napa Cabbages

**Figure 1:**
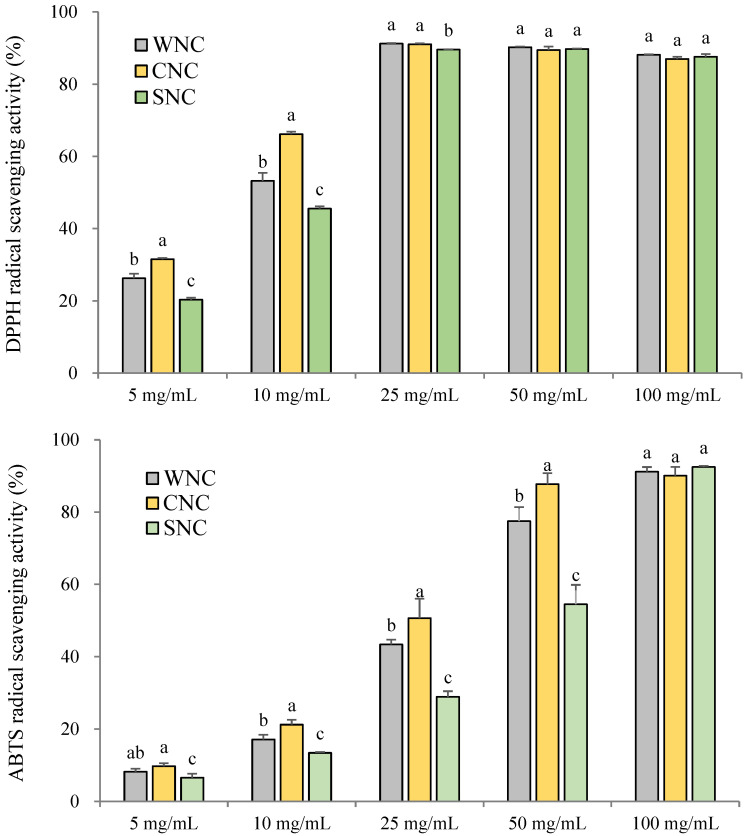
DPPH and ABTS radical scavenging activities of pretreated napa cabbage extract. Bars and error bars represent mean and standard deviation, respectively (*n* = 3). ^a–c^ Different letters within the same treated concentration indicate significant differences (*p <* 0.05; one-way ANOVA and Duncan’s multiple range test).

The DPPH and ABTS radical scavenging activities of the pretreated NC samples are shown in Figure 1. DPPH scavenging activity increased in a dose-dependent manner across extract concentrations ranging from 5 to 25 mg/mL. There is no significant increase in activity observed above 25 mg/mL in any of the samples. A similar pattern was observed for ABTS radical scavenging activity, which also increased with extract concentration. Across a range of concentrations, CNC consistently showed the highest antioxidant activity, particularly at 5 and 10 mg/mL for DPPH and from 5 to 50 mg/mL for ABTS (*p* < 0.05). Reyes et al. (2007) reported that the antioxidant capacity of wounded white cabbage was significantly higher than that of fresh cabbage [25]. This enhancement was attributed to mechanical damage, which triggered the activation of enzymes such as phenylalanine ammonia-lyase (PAL), leading to the biosynthesis of phenolic compounds with high antioxidant potential. In addition, wounding stress was shown to induce antioxidant defense systems [25]. These findings suggest that cutting treatment may enhance the antioxidant activity of cabbage.

#### 3.1.3. Glucosinolates and Their Breakdown Products of Pretreated Napa Cabbages

The compositions of glucosinolates and their breakdown products in the pretreated NC samples are shown in Table 2. CNC consistently showed the highest levels of all compounds except for ascorbigen, which was the most abundant in WNC (1.25 ± 0.05 mg/g DW), followed by CNC and SNC (*p* < 0.05). The contents of NGB and glucoerucin were significantly higher in CNC compared to WNC and SNC, with SNC showing the lowest levels in all compounds (*p* < 0.05). Among the compounds analyzed, 4MGB was the most abundant across all samples, with CNC showing higher content (77.65 ± 1.79 mg/g DW) than WNC (73.79 ± 1.17 mg/g DW) and SNC (59.49 ± 4.25 mg/g DW). A similar result was observed for gluconapin, which was highest in CNC (6.32 ± 0.14 mg/g DW), intermediate in WNC, and lowest in SNC (*p* < 0.05). In our previous study, the major glucosinolates identified in kimchi were glucobrassicin and 4MGB, while ascorbigen was the major breakdown product [26,27]. In this study, an expanded analytical approach enabled the identification of broader types of glucosinolates. CNC had the highest levels of glucosinolates and ascorbigen, whereas SNC had the lowest levels compared to the other samples (*p* < 0.05). Loss of these compounds in SNC may occur during salting. This reduction may be attributed to osmotic loss of water-soluble compounds, similar to the observed decrease in TPC. Palani et al. (2016) also reported that salted cabbage exhibited greater glucosinolate degradation and loss than fresh cabbage, and that glucosinolate content continued to decline over the storage period [28]. The results suggest that NC pretreatment has an influence on the retention of health-promoting glucosinolates and their breakdown products prior to kimchi fermentation.

### 3.2. Change in Kimchi Quality During Storage at Different Conditions

#### 3.2.1. Change in pH During Kimchi Storage and Optimal Fermentation Period

Changes in the pH during kimchi fermentation at 4 °C and 15 °C are shown in Table 3. Fermentation progress was monitored using the pH as an indicator to determine the optimal ripening period of kimchi. At both temperatures, the initial pH of non-fermented kimchi was 5.57 ± 0.01, which is consistent with the typical pH range for fresh samples (pH > 5.0). In many previous studies, the optimal fermentation period of kimchi has been defined as the stage when the pH reaches between 4.0 and 4.5, and a pH value below 4.0 is generally considered indicative of over-ripening or excessive fermentation [29,30,31,32]. In this study, the pH of kimchi gradually decreased during storage, reaching 4.32 ± 0.01 after 47 days at 4 °C, corresponding to the optimal fermentation stage (OFK04, pH 4.0–4.5). Continued storage led to an excessive fermentation state, with the pH dropping below 4.0 by day 168 (EFK04, pH 3.98 ± 0.01). In contrast, fermentation progressed more rapidly at 15 °C. The pH decreased to 4.36 ± 0.00—within the optimal range—after only 3 days (OFK15) and further declined to 3.89 ± 0.00 by day 14 (EFK15), indicating excessive fermentation. These findings suggest that kimchi stored at 15 °C ferments approximately 15 times faster than at 4 °C in terms of reaching the optimal pH range.

#### 3.2.2. TPC and LAB Counts of Kimchi Under Different Storage Temperatures

Changes in the TPC and log CFU of *Leuconostoc* spp. and *Lactobacillus* spp. during kimchi fermentation are shown in Table 4. As fermentation progressed from the NFK to the optimal (OFK04 and OFK15) and excessive fermentation stages (EFK04 and EFK15), distinct shifts were observed in both chemical and microbial profiles.

The TPC in kimchi was significantly higher in fermented samples stored at 15 °C (OFK15: 3.53 ± 0.13; EFK15: 3.64 ± 0.06 mg GAE/g DW) compared to NFK, OFK04, and EFK04 (*p* < 0.05). In contrast, excessive fermentation kimchi stored at 4 °C (EFK04) had the lowest TPC (2.33 ± 0.11 mg GAE/g DW). Briefly, at 4 °C, the TPC did not significantly change during the optimal fermentation period (OFK04) but showed a significant decrease in the excessively fermented sample stored for over five months (EFK04). In contrast, in the 15 °C samples, which underwent relatively short-term fermentation, there was no significant difference in the TPC between OFK15 and EFK15. Ryu et al. (2019) reported that the TPC of kimchi gradually increased during refrigerated storage up to 4 weeks [33], while Thilakarathna et al. (2021) observed a continuous increase in the TPC for up to 5 months, followed by a decrease thereafter [34], indicating potential degradation or transformation of polyphenols during prolonged low-temperature fermentation. Therefore, the findings of this study suggest that the TPC of kimchi during fermentation may be more strongly influenced by the period of storage than by fermentation temperature, and that storage at 15 °C may help prevent TPC degradation by reaching the OFK and EFK stages within a shorter period compared to storage at 4 °C.

Regarding microbial composition, *Leuconostoc* counts peaked in OFK15 (8.484 ± 0.061 log CFU/mL), followed closely by OFK04 (8.166 ± 0.045 log CFU/mL), and then declined markedly in the EFK04 (3.653 ± 0.017 log CFU/mL) and EFK15 (6.960 ± 0.117 log CFU/mL).

Similarly, *Lactobacillus* spp. counts were highest in OFK15 (8.988 ± 0.030 log CFU/mL), followed by OFK04 and EFK15, while significantly lower levels were observed in NFK and EFK04 (*p* < 0.05). Thilakarathna et al. reported that total bacterial counts during storage at 4 °C reached a maximum at 1 month (approximately 7 × 10^8^ CFU/g) and decreased thereafter, showing a 40-fold reduction by 6 months (approximately 2 × 10^6^ CFU/g). In addition, the relative abundance of *Lactobacillales* decreased from approximately 15% at 1 month to below 5% in 5 months [34]. Laksana et al. (2022) reported that when kimchi was stored at 5 °C and 20 °C, LAB counts peaked within 10 days [35]. After that, LAB counts decreased in the kimchi stored at 5 °C, whereas they were maintained in the kimchi stored at 15 °C [35]. Jung et al. (2024) reported that when kimchi was stored at 4 °C and 15 °C for 4 weeks, the LAB count increased most significantly at 15 °C after 1 week, reaching 8.49 ± 0.11 log CFU/mL, indicating a higher LAB cell count at 15 °C compared to 4 °C [9]. The results of this study indicate that OFK15 had significantly higher LAB counts and polyphenol content than OFK04 and that long-term cold storage at 4 °C may lead to a decrease in both LAB populations and polyphenols.

#### 3.2.3. DPPH and ABTS Radical Scavenging Activities of Kimchi Under Different Storage Temperatures

**Figure 2:**
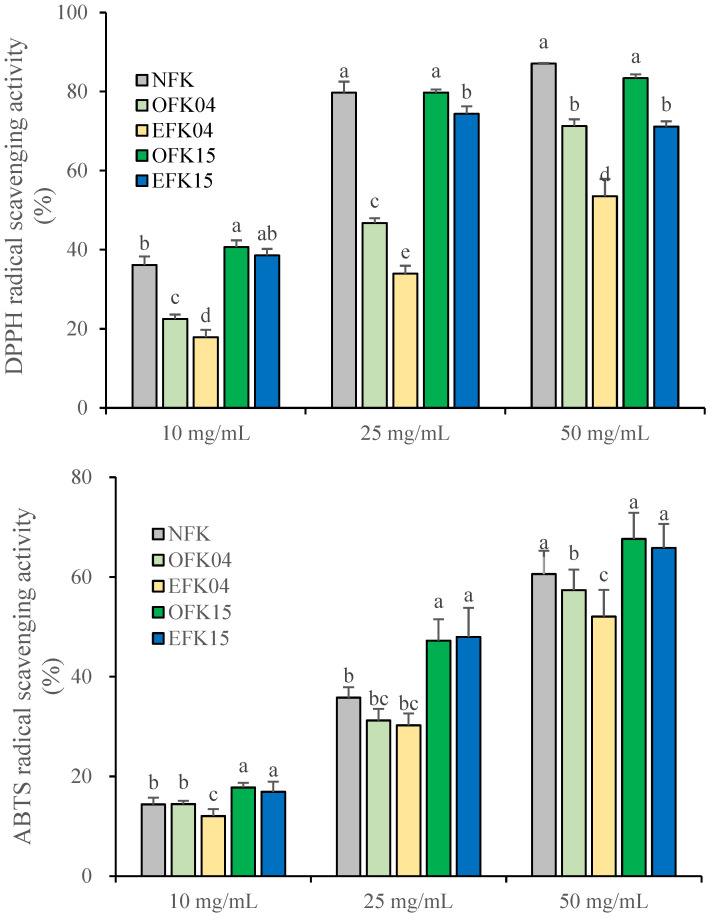
DPPH and ABTS radical scavenging activities of kimchi extract under different storage temperatures. Bars and error bars represent mean and standard deviation, respectively (*n* = 3). ^a–e^ Different letters within the same treated concentration indicate significant differences (*p <* 0.05; one-way ANOVA and Duncan’s multiple range test).

DPPH and ABTS radical scavenging activities of fermented kimchi samples are shown in Figure 2. The DPPH assay revealed a concentration-dependent increase in scavenging activity across all samples. At 10 mg/mL, OFK15 had the highest activity, followed by OFK15 and NFK, whereas EFK04 showed the lowest radical scavenging activity (*p* < 0.05). At 25 and 50 mg/mL, both NFK and OFK15 had significantly higher radical scavenging activity (about 80%) than other groups, while EFK04 was lower (about 30%, *p* < 0.05).

In the ABTS assay, similar results were observed as with the DPPH assay. All the concentrations, OFK had higher radical scavenging activity than EFK regardless of storage temperature (*p* < 0.05). These results collectively indicate that controlled fermentation at 15 °C improves the antioxidant profile of kimchi, while excessive fermentation at 4 °C may result in the degradation or inactivation of key antioxidant compounds.

Ryu et al. (2019) reported that DPPH and ABTS radical scavenging activities decreased during refrigerated storage of kimchi for up to 4 weeks [33]. Similarly, Kim et al. (2014) observed a decline in antioxidant indicators such as DPPH under over-ripened conditions (pH 3.8) during kimchi storage at 5 °C [31]. Hunaefi et al. (2013) reported that the dominance of specific LAB strains may influence the content of antioxidant compounds, leading to increased antioxidant activity during fermentation [36]. Briefly, fermentation may induce structural breakdown of plant cell walls as pH decreases, facilitating the enzymatic release of bound phenolic constituents and promoting the liberation and/or synthesis of various bioactive compounds, thereby enhancing antioxidant activity [36,37]. These findings indicate that controlled fermentation periods at 15 °C improves the antioxidant profile of kimchi, whereas prolonged or excessive fermentation at 4 °C may result in the degradation of antioxidant compounds.

#### 3.2.4. Glucosinolates and Their Breakdown Products of Kimchi Under Different Storage Temperatures

The compositions of glucosinolates and their breakdown products in the fermented kimchi samples are presented in Table 5. These compounds varied significantly depending on the fermentation conditions. In the NFK, major glucosinolates—including NGB (4.18 ± 0.29 mg/g DW), 4MGB (33.32 ± 2.00 mg/g DW), and glucobrassicin (6.26 ± 0.41 mg/g DW)—were present at the highest levels (*p* < 0.05). In contrast, these compounds were not detected in the excessively fermented samples (EFK04 and EFK15), indicating near-complete degradation during prolonged fermentation. Among the fermented samples, OFK15 retained low levels of NGB (0.96 ± 0.04 mg/g DW) and 4MGB (13.54 ± 0.48 mg/g DW), whereas OFK04 contained only 4MGB (3.73 ± 0.43 mg/g DW). Glucoraphanin was detected only in NFK and OFK15 and was significantly reduced in the latter (2.48 ± 0.19 mg/g DW, *p* < 0.05). Breakdown products such as ascorbigen and sulforaphane showed different patterns. Ascorbigen increased from 0.81 ± 0.02 mg/g DW in NFK to 2.13–2.61 mg/g DW in the fermented samples, reaching its highest level in EFK15 (2.61 ± 0.08 mg/g DW). In contrast, sulforaphane was detected only in OFK04 (0.30 ± 0.07 mg/g DW).

Palani et al. (2016) reported that glucosinolates such as GB, 4MGB, and glucoraphanin decreased continuously during the fermentation of white cabbage, while the levels of ascorbigen and other breakdown products increased in the opposite trend [28]. However, as fermentation progressed, ascorbigen levels also declined. GB is a major precursor of ascorbigen, and Palani et al. observed a sharp increase in ascorbigen as glucobrassicin levels decreased during fermentation, which is consistent with the findings of this study [28]. Similarly, Peñas et al. (2010) reported that fermentation of raw cabbage resulted in a sharp decline in glucosinolate content and simultaneously production of ascorbigen, regardless of the salinity level [38]. They further reported that ascorbigen levels remained relatively stable up to 1 month but decreased markedly with extended fermentation, falling to less than half of the 1-month level by 3 months [38]. These findings support the results of this study, indicating that under long-term storage at 4 °C, ascorbigen content does not necessarily peak at the point of optimal pH and may be largely degraded during over-ripening. In contrast, short-term fermentation at 15 °C resulted in a higher peak level of ascorbigen, which was well retained even under excessive fermentation conditions. Ascorbigen is the major glucosinolate breakdown product found in fermented cabbages such as kimchi and sauerkrauts [17,27,39], and it is one of the metabolites from glucosinolates that enhances immune systems and anticarcinogens [6,39]. Considering the health-promoting effects of ascorbigen, such as its anti-inflammatory and anticancer properties, these findings suggest that short-term fermentation may be more favorable for enhancing the functional health benefits of kimchi.

Therefore, these results indicate that controlled short-term fermentation at 15 °C can partially preserve glucosinolates while enhancing ascorbigen levels, whereas prolonged fermentation leads to substantial degradation of glucosinolates and their breakdown products.

## 4. Conclusions

Pretreatment of napa cabbage (WNC, CNC, and SNC) affected total phenolic content, antioxidant activity, and glucosinolate levels, with CNC generally showing the highest values. The results of this study highlight that fermentation period plays a more critical role than fermentation temperature in determining the functional quality such as TPC, radical scavenging activities, LAB counts, and glucosinolates of kimchi during storage. Short-term fermentation at 15 °C (OFK15: 3 days; EFK15: 14 days) led to higher levels of TPC, ascorbigen, and LAB counts, as well as enhanced antioxidant activities, compared to long-term fermentation at 4 °C (OFK04: 47 days; EFK04: 168 days). In contrast, prolonged storage at 4 °C was associated with the degradation of glucosinolates and a decline in microbial viability. Unlike the traditional long-term fermentation process historically used to achieve optimal quality, short-term fermentation at moderate temperature can yield kimchi with superior functional properties in a much shorter time. This approach may offer advantages for the kimchi industry by enabling shorter fermentation periods while enhancing the functional quality of the final product. This study focused on changes in functional components under different fermentation conditions but did not address consumer preferences, texture, or microbiological safety. Further research in diverse areas is needed to advance innovative kimchi production technologies that are both safe and beneficial for consumers.

## Figures and Tables

**Table 1:** Total polyphenol content and lactic acid bacteria counts in napa cabbage with different pretreatments.

Sample	Total Polyphenol Content (mg GAE/g DW)	*Leuconostoc* spp. (Log CFU)	*Lactobacillus* spp. (Log CFU)
WNC	3.70 ± 0.18 ^a^	3.05 ± 0.13	3.15 ± 0.09
CNC	3.80 ± 0.05 ^a^	ND	ND
SNC	2.81 ± 0.11 ^b^	ND	3.12 ± 0.05

WNC: whole napa cabbage, CNC: cut napa cabbage, SNC: salted napa cabbage, and ND: not detected. Values are means ± standard deviations (*n* = 3). ^a,b^ Different superscripts within the same columns indicate significant differences (*p <* 0.05; one-way ANOVA and Duncan’s multiple range test).

**Table 2:** Glucosinolates and their breakdown products in napa cabbage with different pretreatments.

	Glucosinolates (mg/100 g)								Breakdown Products (mg/100 g)	
Sample	Neoglucobrassicin	4-Methoxyglucobrassicin	Glucobrassicin	Glucoraphenin	Glucoerucin	Glucoraphasatin	Progoitrin	Gluconapin	Ascorbigen	Sulforaphane
WNC	9.61 ± 0.01 ^b^	73.79 ± 1.17 ^a^	12.57 ± 0.16 ^a^	ND	2.39 ± 0.10 ^b^	ND	14.50 ± 0.60 ^a^	5.95 ± 0.38 ^a^	1.25 ± 0.05 ^a^	ND
CNC	12.22 ± 0.30 ^a^	77.65 ± 1.79 ^a^	12.84 ± 0.22 ^a^	ND	2.85 ± 0.20 ^a^	ND	15.07 ± 0.88 ^a^	6.32 ± 0.14 ^a^	1.19 ± 0.03 ^a^	ND
SNC	8.09 ± 0.63 ^c^	59.49 ± 4.25 ^b^	10.47 ± 0.81 ^b^	ND	1.94 ± 0.18 ^c^	ND	10.96 ± 1.37 ^b^	5.26 ± 0.36 ^b^	0.51 ± 0.02 ^b^	ND

WNC: whole napa cabbage, CNC: cut napa cabbage, SNC: salted napa cabbage, and ND: not detected. Values are means ± standard deviations (*n* = 3). ^a–c^ Different superscripts within the same columns indicate significant differences (*p <* 0.05; one-way ANOVA and Duncan’s multiple range test).

**Table 3:** Fermentation stages of kimchi under different storage temperatures.

	Expected pH	4 °C		15 °C	
		pH	Storage Weeks (Days)	pH	Storage Weeks (Days)
Non-fermented kimchi	>5	5.57 ± 0.01	0	5.57 ± 0.01	0
Optimally fermented kimchi	4.0~4.5	4.32 ± 0.01	7 (47)	4.36 ± 0.0	1 (3)
Excessively fermented kimchi	<4.0	3.98 ± 0.01	24 (168)	3.89 ± 0.0	3 (14)

Values are means ± standard deviations (*n* = 3).

**Table 4:** Total polyphenol content and lactic acid bacteria counts in kimchi under different storage temperatures.

Sample	Total Polyphenol Content (mg GAE/g DW)	*Leuconostoc* spp. (Log CFU/mL)	*Lactobacillus* spp. (Log CFU/mL)
NFK	2.98 ± 0.02 ^b,c^	5.497 ± 0.127 ^d^	5.643 ± 0.071 ^e^
OFK04	3.07 ± 0.06 ^b^	8.166 ± 0.045 ^b^	7.285 ± 0.034 ^c^
EFK04	2.33 ± 0.11 ^d^	3.653 ± 0.017 ^e^	5.976 ± 0.166 ^d^
OFK15	3.53 ± 0.13 ^a^	8.484 ± 0.061 ^a^	8.988 ± 0.030 ^a^
EFK15	3.64 ± 0.06 ^a^	6.960 ± 0.117 ^c^	7.693 ± 0.087 ^b^

NFK: non-fermented kimchi, OFK04: optimally fermented kimchi stored at 4 °C, EFK04: excessively fermented kimchi stored at 4 °C, OFK15: optimally fermented kimchi stored at 15 °C, and EFK15: excessively fermented kimchi stored at 15 °C. Values are means ± standard deviations (*n* = 3). ^a–e^ Different superscripts within the same columns indicate significant differences (*p <* 0.05; one-way ANOVA and Duncan’s multiple range test).

**Table 5:** Glucosinolates and their breakdown products in kimchi under different storage temperatures.

Glucosinolates (mg/100 g)									Breakdown Products (mg/100 g)	
Sample	Neoglucobrassicin	4-Methoxyglucobrassicin	Glucobrassicin	Glucoraphenin	Glucoerucin	Glucoraphasatin	Progoitrin	Gluconapin	Ascorbigen	Sulforaphane
IPK	4.18 ± 0.29 ^a^	33.32 ± 2.00 ^a^	6.26 ± 0.41	3.96 ± 0.13	1.59 ± 0.10	130.65 ± 9.37	5.02 ± 0.51	3.07 ± 0.33	0.81 ± 0.02 ^d^	ND
OFK4	ND	3.72 ± 0.43 ^c^	ND	ND	ND	ND	ND	ND	2.13 ± 0.33 ^b^	0.30 ± 0.07
EFK4	ND	ND	ND	ND	ND	ND	ND	ND	0.39 ± 0.02 ^e^	ND
OFK15	0.96 ± 0.04 ^b^	13.55 ± 0.48 ^b^	1.23 ± 0.04 *	2.48 ± 0.19 *	ND	31.56 ± 2.17 *	1.37 ± 0.13 *	ND	2.59 ± 0.11 ^a^	ND
EFK15	ND	1.55 ± 0.03 ^d^	ND	ND	ND	ND	ND	ND	2.61 ± 0.08 ^a^	ND

NFK: non-fermented kimchi, OFK04: optimally fermented kimchi stored at 4 °C, EFK04: excessively fermented kimchi stored at 4 °C, OFK15: optimally fermented kimchi stored at 15 °C, EFK15: excessively fermented kimchi stored at 15 °C, and ND: not detected. Values are means ± standard deviations (*n* = 3). ^a–e^ Different superscripts within the same columns indicate significant differences (*p <* 0.05; one-way ANOVA and Duncan’s multiple range test). * Significant difference (*p* < 0.05; Student’s independent *t*-test).

## Data Availability

The original contributions presented in the study are included in the article, and further inquiries can be directed to the corresponding author.

## Supplementary Materials

The following supporting information can be downloaded at: https://www.mdpi.com/article/10.3390/foods14162826/s1, Table S1: UPLC-MS/MS analysis conditions. Table S2: Parameters for analysis of glucosinolates and their breakdown products by UPLC-MS/MS MRM.
