# Supplementary material for: Effect of Fermentation Conditions on Functional Quality of Napa Cabbage Kimchi
Source: Foods. 2025 Aug 15;14(16):2826. doi: 10.3390/foods14162826 (PMC12385461; doi:10.3390/foods14162826)
Supplement: Supplementary file 1 [file foods-14-02826-s001.zip › foods-3807629-supplementary.pdf]

Supplementary materials

Table S1. UPLC-MS/MS analysis conditions.

Parameter	Condition
Solvent A	0.1% formic acid in distilled water
Solvent B	0.1% formic acid in acetonitrile
Column oven	40 °C
Flow rate	0.3 mL/min
Injection volume	2 µL
Gradient	0-0.5 min, 0% B; 0.5-8.5 min, 100% B; 8.5-10.5 min, 100% B; 10.5-10.8 min, 0% B; 10.8-13 min, 0% B
Ionization method	Electrospray ionization (negative ion mode)
Capillary voltage	3 kV
Cone voltage	50 V
Desolvation temperature	350 °C
Source temperature	150 °C
Desolvation gas	1000 L/h
Cone gas	150 L/h
Collision energy	40 V
Software	MassLynx V.4.1. (Waters)

Table S2. Parameters for analysis of glucosinolates and their breakdown products by UPLC-MS/MS MRM.

Compounds	Retention time (min)	Formula	Molecular weight	Ionization	MRM transition	
					Precursor ion (m/z)	Product ion (m/z)
Glucosinolate						
Glucoraphanin	2.33	$C_{12}H_{23}NO_{10}S_3$	437.507	$[M-H]^-$	436	97
Glucoraphenin	2.66	$C_{12}H_{21}NO_{10}S_3$	435.491	$[M-H]^-$	434	97
Glucoerucin	5.81	$C_{12}H_{23}NO_9S_3$	421.507	$[M-H]^-$	420	97
Glucoraphasatin	5.91	$C_{12}H_{21}NO_9S_3$	419.507	$[M-H]^-$	418	97
Glucobrassicin	6.10	$C_{16}H_{20}N_2O_9S_2$	448.468	$[M-H]^-$	447	97
4-Methoxyglucobrassicin	6.65	$C_{17}H_{22}N_2O_{10}S_2$	478.494	$[M-H]^-$	477	97
Breakdown product						
Ascorbigen	6.16	$C_{15}H_{15}NO_6$	305.283	$[M+H]^+$	306	130
Sulforaphane	6.45	$C_6H_{11}NOS_2$	177.288	$[M+H]^+$	178	72
Indole-3-carboxaldehyde	6.86	C_9H_7NO	145.158	$[M+H]^+$	146	118
